# The risk of cognitive impairment associated with hearing function in older adults: a pooled analysis of data from eleven studies

**DOI:** 10.1038/s41598-018-20496-w

**Published:** 2018-02-01

**Authors:** Jing Yuan, Yu Sun, Shuping Sang, Jessica Huynh Pham, Wei-Jia Kong

**Affiliations:** 1 Department of Otorhinolaryngology, Union Hospital, Tongji Medical College, Huazhong University of Science and Technology, Wuhan, Hubei 430022 PR China; 2 School of Medicine, Yunnan University, Kunming, Yunan 650031 PR China; 3 School of Medicine, Case Western Reserve University, Cleveland, Ohio, 44106 United States

## Abstract

Impaired hearing and cognition are disabling conditions among older adults. Research has presented inconsistent conclusions regarding hearing impairment posing a risk for cognitive impairment. We aimed to assess this from published evidence via searching PubMed and Embase, from the inception of the databases indexed to December 2, 2016. For those high-quality studies retrieved, relative risk (RR) and 95% confidence intervals (CIs) were combined to estimate the risk of cognitive impairment. Eleven cohort studies were included in the present study. Pooled results found that elderly people with disabled peripheral and central hearing function had a higher risk of cognitive impairment (for moderate/severe peripheral hearing impairment: RR = 1.29, 95% CI: 1.04–1.59 during a follow-up ≤6 years. RR = 1.57, 95% CI: 1.13–2.20 during a follow-up >6 years; for severe central hearing impairment, RR = 3.21, 95% CI: 1.19–8.69) compared to those with normal hearing function. We also recorded a dose-response trend for cognitive impairment as hearing thresholds rose. No evident bias from potential confounding factors was found with one exception: the length for clinical follow-up. Although results are preliminary because qualifying studies were few, statistical findings were consistent with older people identified as having greater levels of hearing loss, having a corresponding higher risk of cognitive impairment.

## Introduction

Age-related hearing loss (ARHL), a hearing impairment caused by aging and neurodegeneration, is characterized in older adults as difficulty in understanding speech and detecting sound^1^. Depending upon the auditory pathways involved, ARHL can be categorized as peripheral ARHL or central ARHL, and the clinical manifestations are often mixed^2^. ARHL has become a major sensory condition, within the world’s rapidly growing aging population^1^. American prevalence rates showed that hearing impairment affected 29.3% of the population at age 60 to 69 years^3^ and the percentage was increased to 63.1% in people aged 70 years or over^4^. Surveys from other countries also revealed the leading role of ARHL among disabling conditions associated with older demographics^5,6^. Apart from reduced hearing sensitivity and speech understanding, ARHL has a series of consequences including reduced ability to detect and localize safety and/or warning alarms^1^, and compromised communication efficiency linked to comorbid psychosocial issues such as social isolation and depression^7^. Although there are numerous rehabilitative alternatives for ARHL, patients and their families may not always seek such options; therefore, the condition is largely underestimated, especially during an early stage of ARHL^2^. Patients generally fail to obtain sufficient screening and intervention probably because they regard ARHL as simply a part of entering their senior years, unaware of its potentially far-reaching consequences^1,8^.

However, ARHL may be widely associated with neurodegenerative, functional, physical, and psychosocial impairment^9^. For instance, ARHL serves as one of the substantial markers of frailty (a nonspecific state of vulnerability, decreased physiological reserve, and reduced resistance to stressors^10^) in older age with adverse outcomes like cognitive impairment^7^. Cognitive impairment affects many domains such as: memory, attention, executive function, perception, and semantic knowledge, which also constitute some of the primary targets of dementia and Alzheimer’s disease (AD)^11^. Epidemiological evidence supports the high possibility of cognitive impairment progressing into dementia and AD^12^, both of which have rapidly-increasing prevalence with age and the absence of disease-modifying treatment^2,8,13^. Prevention of dementia and AD has become a public health priority due to its irreversibility and its burden upon individuals, families, and society^14^.

An increasing number of findings have suggested an association between ARHL and cognitive impairment, indicating that ARHL may be a potential early marker of AD^2,7^. Thus, collecting evidence from observational studies could be an important initiative to assess whether ARHL serves as a modifiable factor among the strategies of preventing dementia. An initial review appeared to provide some support for the relationship between ARHL and cognitive impairment; however, it also identified some studies which argued against this correlation^15–19^. More recent studies^14,20–22^ have emerged. They applied more recognized measures to evaluate the auditory and mental status of various populations. However, research with negative results^23,24^ has prevented us from definitively concluding that hearing function is connected to the risk of cognitive impairment during the later life of adults.

Previous systematic reviews and meta-analyses^25,26^ have offered some perspectives demonstrating that hearing impairment and cognitive problems are associated. To our knowledge, the first meta-analysis to explore hearing loss and cognitive function^25^, without putting restrictions on age or methodology when retrieving studies, concluded that individuals with hearing loss had worse cognitive performance. A recent study^26^ gathered some prospective cohort studies to support the conclusion that hearing impairment increases the risk of both cognitive disorders and AD, though it analyzed only four heterogeneous studies. Categories of hearing function and possible confounding factors were not explored in either study.

The present meta-analysis of cohort studies was undertaken to explore research inconsistencies regarding ARHL and cognitive impairment, statistically. By including more recent data, we appraised the hearing function-cognition relationship in older adults and its dose-response trend with more participants. We also incorporated both peripheral and central hearing function as independent variables to carry out an analysis for the risk of cognitive impairment by category. Additionally, the present study considered potential confounding factors such as race and sex in meta-regression because, for example, melanin and estrogen may work in the pathogenesis of ARHL^27,28^. Follow-up durations were considered because both ARHL and cognitive impairment are conditions that deteriorate with age^2^. Apart from the hearing function-cognition relationship, we also analyzed the initial findings from these retrieved studies about the effect of hearing aid use on the incidents of cognitive impairment.

## Results

### Study selection, characteristics and quality assessment

**Figure 1:**
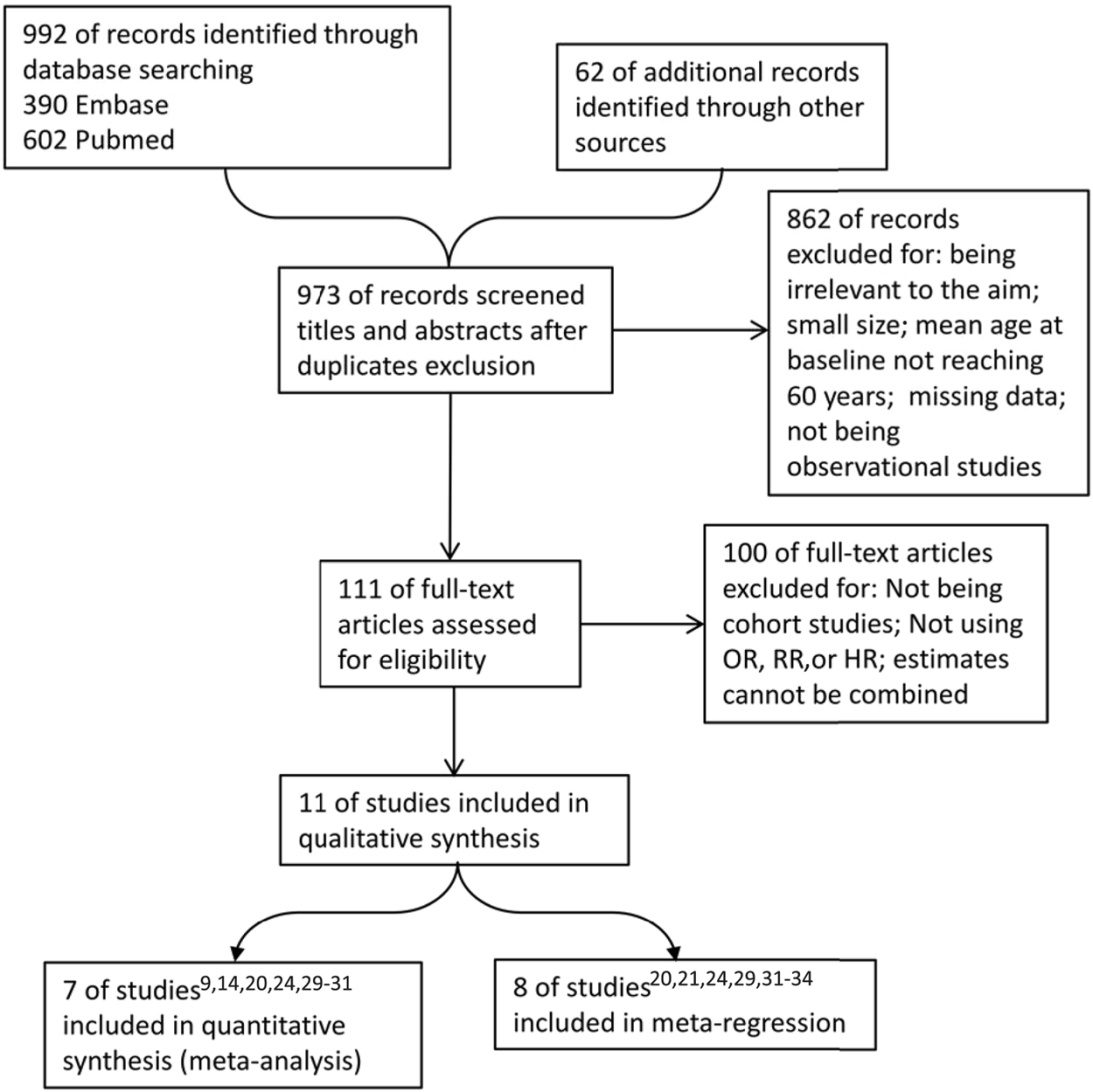
PRISMA flow chart of study selection. PRISMA = Preferred Reporting Items for Systematic Reviews and Meta-analyses. OR = odds ratio, RR = relative risk, HR = hazard ratio.

Figure 1 presents the flow chart used for determining eligible research. Through databases and manual searching, 973 studies were found after subtracting duplicates. Investigators next went through titles and abstracts to exclude the studies that had missing data, a small sample size (n < 100), a mean age at baseline below 60 years, or studies that were non-observational and not consistent with our aim. Full text of the remaining 111 were further analyzed. Of these, those with estimates that could not be combined, that were not cohort-designed, and those which used estimates other than odds ratio (OR), relative risk (RR), and hazard ratio (HR), were also omitted at this stage. Finally, eleven studies^9,14,20,21,24,29–34^ were included, seven of which^9,14,20,24,29–31^ were combined in the following meta-analyses. Eight studies^20,21,24,29,31–34^ were analyzed in meta-regression.

Table 1 summarizes studies that were selected for analysis. Eleven related studies were enrolled into the present study. All prospective cohort studies included participants across Europe^32^, North America^14,20,21,29–31,33^, Oceania^9,24^, and Asia^34^. All cohorts, except for one^21^, included both genders. Some studies evaluated hearing function with insurance records^32^ and self-reporting^33,34^, and the rest used pure-tone audiometry. Five cohorts were followed-up ≤6 years^20,21,24,31,32^. Cognitive status was qualified or quantified by the Mini-Mental State Examination (MMSE) and its revised version^9,20,21,24,29,31,33,34^, or the Diagnostic and Statistical Manual (DSM) and the National Institute of Neurological and Communicative Disorders and Stroke and the Alzheimer’s Disease and Related Disorders Association (NINCDS-ADRDA)^14,30,33^. All studies were multi-adjusted of covariates with three exceptions^24,31,34^. Quality assessment is presented in Supplementary Table S1 and all included studies scored more than five stars on the Newcastle-Ottawa Scale (NOS).

**Table 1:** Characteristics of included studies.

Studies	Country (Ethnicity)	Mean age at baseline (SD)	Participant numbers	Female (%)	Event numbers	Maximum follow-up years	Hearing function evaluations	Instruments qualifying/quantifying cognitive status
Deal JA^29^	US (White and Black)	75.5 (3.0)	1,889	52.7	229	9	Pure-tone audiometry	3MS score
Fritze T^32^	Germany (NA)	≥ 65	154,783	NA	14,602	6	ICD-10	ICD-10
Gates GA^31^	US (NA)	72 (63–95)^a^	1,662/1,026^b^	60.2	41	6	Pure-tone audiometry, SSI-ICM	MMSE score
Gates GA^30^	US (NA)	79.6 (5.2)	274	62.8	21	2.2 (0.8–4)^a^	SSI-ICM	CASI score, DSM-IV, and NINCDS-ADRDA
Gurgel RK^33^	US (NA)	75.5 (6.9)	4,463	60	574	5.8 (4.2)^c^	Self-report interview	3MS score, DSM-III-R, and NINCDS-ADRDA
Hong T^24^	Australia (NA)	68.2 (7.9)^d^	1,638^d^	43.7^d^	NA	10	Pure-tone audiometry	MMSE blind
Karpa MJ^9^	Australia (NA)	66.6 (9.3)	2,815	56.7	NA	9	Pure-tone audiometry	MMSE score
Lin FR^14^	US (Black, White and other)	63.6 (12.8)	639	43.7	58	11.9^e^	Pure-tone audiometry	DSM-III-R, and NINCDS-ADRDA
Lin FR^20^	US (White and Black)	77.4 (2.8)^f^	1,626	52.1^f^	609	6	Pure-tone audiometry	3MS score
Lin MY^21^	US (Black excluded)	76.1 (NA)	5,345	100	NA	4.4 (NA)^c^	Pure-tone audiometry	3MS score
Lyu J^34^	South Korea (Asian)	71.1 (4.9)	1,759	52.6	501	6	Self-report	Korean MMSE

Abbreviations: 3MS = Modified Mini-Mental State Examination, CASI = Cognitive Abilities Screening Instrument, DSM = Diagnostic and Statistical Manual, ICD = International Classification of Diseases, MMSE = Mini-Mental State Examination, NA = not available, NINCDS-ADRDA = National Institute of Neurological and Communicative Disorders and Stroke and the Alzheimer’s Disease and Related Disorders Association, SD = standard deviation, SSI-ICM = Synthetic Sentence Identification with Ipsilateral Competing Message, UK = United Kingdom, US = United States. ^a^Mean (range), ^b^peripheral hearing test/central hearing test, ^c^mean (SD), ^d^data from hearing impairment cohort and controls, ^e^median, ^f^data from the whole cohort.

## Overall Results

**Figure 2:**
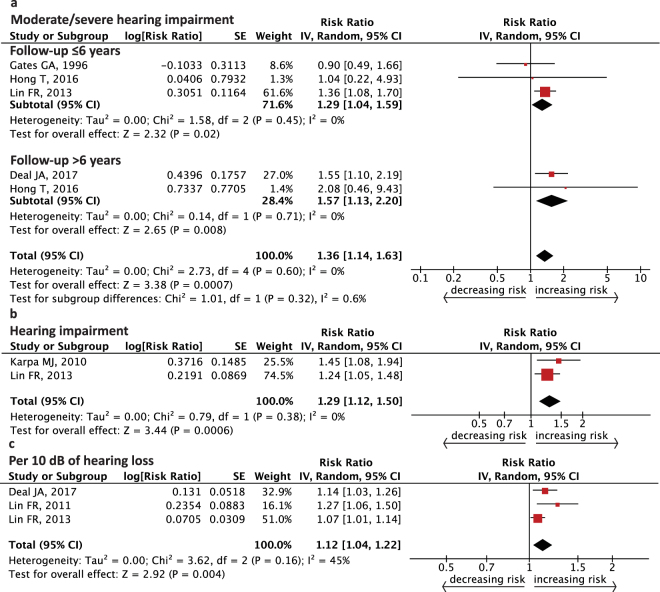
Forest plot showing the risk of incident cognitive impairment from peripheral auditory function. (**a**) Pooled relative risk from moderate/severe hearing impairment (PTA >40 dB HL). (**b**) Pooled relative risk from hearing impairment (PTA >25 dB HL). (**c**) Pooled relative risk per 10 dB of hearing loss. CI = confidence interval, dB HL = decibels hearing level, IV = inverse variance, PTA = pure-tone average, SE = standard error.

Figure 2 shows the cumulative risk for cognitive impairment from peripheral hearing functioning of the better ear at baseline. When the hearing threshold was greater than 40 decibels hearing level (dB HL) for the pure-tone average (PTA) at 0.5, 1, 2, and 4 kHz (subjects identified as having moderate/severe hearing impairment^29^), the risk of cognitive impairment in older subjects increased 29–57% compared to those with normal hearing (follow-up ≤6 years, RR = 1.29, 95% confidence interval (CI): 1.04–1.59; follow-up >6 years, RR = 1.57, 95% CI: 1.13–2.20, Fig. 2a). Older people were also at a risk of cognitive impairment when the hearing level was abnormal (subjects with PTA > 25 dB HL identified to have hearing impairment^35^; RR = 1.29, 95% CI: 1.12–1.50, Fig. 2b). In seniors, the estimated incidence of cognitive impairment had a 12% increase when PTA was modeled continuously (for every 10 dB increase in hearing loss, RR = 1.12, 95% CI: 1.04–1.22, Fig. 2c). No significant heterogeneity was seen among the studies except in Fig. 2c (in subgroup of follow-up ≤6 years, *I*^2^ = 0%, *P* = 0.45, in subgroup of follow-up >6 years, *I*^2^ = 0%, *P* = 0.71, Fig. 2a; *I*^2^ = 0%, *P* = 0.38, Fig. 2b; *I*^2^ = 45%, *P* = 0.16, Fig. 2c).

**Figure 3:**
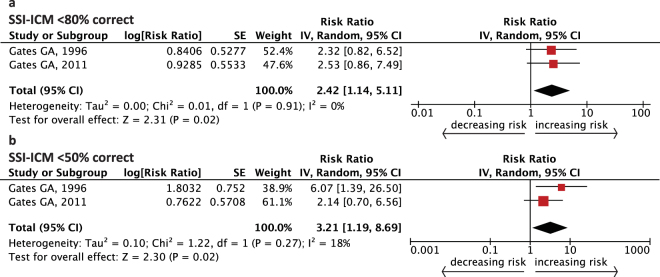
Forest plot showing the risk of incident cognitive impairment from one central auditory processing test. Combined relative risk from (**a**) moderate impaired central auditory processing (SSI-ICM <80% correct) and (**b**) severe impaired central auditory processing (SSI-ICM <50% correct). CI = confidence interval, IV = inverse variance, SE = standard error, SSI-ICM = Synthetic Sentence Identification with Ipsilateral Competing Message.

Figure 3 demonstrates the pooled RR of cognitive impairment when participants showed abnormality in one of the central auditory processing (CAP) tests at baseline. Here, central auditory function was obtained by Synthetic Sentence Identification with Ipsilateral Competing Message (SSI-ICM). SSI-ICM <80% correct is considered consistent with central auditory dysfunction (CAD) based on this test’s normative data^30,31^. The RR for incidence of cognitive impairment in older people was 2.42 in the moderate CAD group compared with the normal function group (SSI-ICM < 80% correct, RR = 2.42, 95% CI: 1.14–5.11, Fig. 3a). When CAP was severely abnormal on the SSI-ICM test (<50% correct), the risk elevated to 3.21 (RR = 3.21, 95% CI: 1.19–8.69, Fig. 3b). No obvious heterogeneity was detected among these studies (*I*^2^ = 0%, *P* = 0.91, Fig. 3a; *I*^2^ = 18%, *P* = 0.27, Fig. 3b).

**Figure 4:**
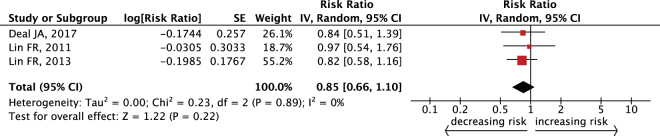
Forest plot showing the effect of hearing aid use on incident cognitive impairment. CI = confidence interval, IV = inverse variance, SE = standard error.

Figure 4 demonstrates that we recorded no significant reduction in the risk for cognitive impairment among people who were using hearing aids (RR = 0.85, 95% CI: 0.66–1.10). There was no evidence of heterogeneity in this analysis (*I*^2^ = 0%, *P* = 0.89).

## Subgroup and Sensitivity Analysis

Table 2 provides a dose-response association between peripheral hearing loss and cognitive impairment concluded from 3 studies^14,20,29^. Analyses were performed with groups stratified by the severity of hearing loss, evaluated with PTA results of thresholds in the better ear (Categories of ≤25 dB HL, 26–40 dB HL, 41–70 dB HL and >70 dB HL mean normal, mild, moderate, and severe hearing loss, respectively). Relative risk from the studies was combined with the risk ranging from 1.19 (95% CI: 0.96–1.48) to 4.94 (95% CI: 1.09–22.40) across three hearing loss categories.

**Table 2:** Association of severity of peripheral hearing loss with incident cognitive impairment.

PTA level	Studies#	RR (95% CI)	*P*_*heterogeneity*_	*I* ^*2*^
≤25 dB HL	—	1, reference	—	—
26–40 dB HL	3^14,20,29^	1.19 (0.96, 1.48)	0.23	31%
41–70 dB HL	1^14^	3.00 (1.43, 6.30)	—	—
>70 dB HL	1^14^	4.94 (1.09, 22.40)	—	—

Abbreviations: CI = confidence interval, PTA = pure-tone average, RR = relative risk, dB HL = decibels hearing level.

Table 3 suggests the influence of potential confounding factors while assessing the relationship between peripheral hearing loss and cognitive impairment with meta-regression. We recorded no evident bias arising from the ear sides associated with PTA results (better ear, worse ear, one ear, two ears, or nonspecific), ethnicity (mixed cohorts, Black-excluded cohorts, or Asian cohorts), gender (mixed cohorts, male cohorts, or female cohorts), level of adjustment (confounding factors being fully adjusted, partially adjusted, or not adjusted), subjectivity of the hearing measurement (pure-tone audiometry versus self-report or others), and instruments for cognitive status (MMSE or related versions versus others) with one exception: maximum time to follow-up (when cohorts were divided into subgroups that had been followed-up ≤6 years or followed-up >6 years, follow-up durations were a likely source of confounding when analyzing peripheral hearing loss and cognitive impairment, *P* = 0.004). The data for meta-regression were gathered in Supplementary Table S2.

**Table 3:** Association of peripheral hearing loss and incident cognitive impairment (in relation to: the ear sides associated with PTA results, maximum follow-up of each cohort, racial and sexual distributions of each cohort, statistical adjustment, and hearing and cognitive evaluations each cohort had applied).

Variables	Coefficient (95% CI)	Meta-regression *P*
Ear sides (PTA)	−0.14 (−0.36–0.08)	0.188
Maximum follow-up	0.33 (0.13–0.53)	0.004
Ethnicity	−0.02 (−0.66–0.61)	0.942
Gender	0.15 (−0.15–0.44)	0.294
Adjustment	−0.01 (−0.10–0.08)	0.829
Hearing measures	0.61 (−0.75–1.97)	0.345
Cognitive evaluations	−0.35 (−1.44–0.74)	0.496

Abbreviations: CI = confidence interval, PTA = pure-tone average.

Table 4 shows the sensitivity analysis we have performed. Overall estimates remained relatively unchanged when a single study was omitted sequentially in the meta-analysis. A heterogeneity change in *P*_*heterogeneity*_ and *I*^2^ was observed when evaluating the risk of cognitive impairment for every 10 dB increase in hearing loss.

**Table 4:** Sensitivity analyses of included studies. Abbreviations: CI = confidence interval, dB = decibels, RR = relative risk.

One-study-out method	RR from the remaining studies (95% CI)	*P*_*heterogeneity*_	*I* ^*2*^
**Moderate/severe hearing impairment (follow-up ≤6 years)**			
Gates GA, 1996	1.35 (1.08, 1.69)	0.74	0%
Hong T, 2016	1.23 (0.87, 1.73)	0.22	34%
Lin FR, 2013	0.92 (0.52, 1.62)	0.87	0%
**Per 10 dB of hearing loss**			
Deal JA, 2017	1.14 (0.98, 1.33)	0.08	68%
Lin FR, 2011	1.09 (1.03, 1.15)	0.32	1%
Lin FR, 2013	1.17 (1.07, 1.28)	0.31	4%
**Wearing hearing aids**			
Deal JA, 2017	0.86 (0.63, 1.15)	0.63	0%
Lin FR, 2011	0.83 (0.62, 1.10)	0.94	0%
Lin FR, 2013	0.89 (0.61, 1.31)	0.72	0%

## Publication Bias

Publication bias analysis was not applied here due to a limited number of qualifying studies.

## Discussion

Our major findings showed that, after statistical analyses, both peripheral and central hearing dysfunction appear to contribute to the risk of cognitive impairment in the aging population. The overall risk of cognitive impairment increased 29% (follow-up ≤6 years) or 57% (follow-up >6 years) in senior participants who had disabled peripheral hearing function compared with people of normal hearing function. The risk remained significant when adopting the World Health Organization (WHO) standard of hearing impairment or when PTA was modeled continuously. The analysis showed that the association between central hearing dysfunction and cognitive impairment was stronger (RR = 3.21, 95% CI: 1.19–8.69 for SSI-ICM <50% correct; RR = 2.42, 95% CI: 1.14–5.11 for SSI-ICM <80% correct). A dose-response trend was found between peripheral hearing function and cognition. There was no obvious heterogeneity among most studies, excluding the analysis combining RR for every 10 dB increase in hearing loss (Fig. 2c). After analyzing potential confounding factors including: the ear sides associated with PTA results, ethnicity, gender of cohorts, level of adjustment, as well as hearing and cognitive evaluations, the risk of cognitive impairment remained. Specifically, a maximum follow-up dependent association was found in the meta-regression and the subgroup analysis. However, using hearing aids did not significantly lower the risk of cognitive impairment in elderly people.

Previous meta-analyses^25,26^ had collected encouraging findings of the hearing function-cognition relationship, as it applied to cognition domains or the onset of AD. The first meta-analysis^25^ found that individuals with hearing loss had poorer cognitive manifestations whether hearing was treated or untreated. However, this conclusion was based on adults from a wide range of age groups, applying varied measures on hearing and cognitive status, and varied statistical methods. Another meta-analysis^26^ – which gathered limited but robust-appearing data from prospective studies – also chose a generalizing approach to analyze the relationship between hearing loss and AD. These two studies explored neither categories of hearing function nor possible confounding factors.

Our combined estimates were consistent with previous reviews^2,7^, confirming (by statistical analysis) the hearing function-cognition relationship in older people. In addition, we included more recent high-quality cohort studies and explicitly-classified hearing function as an independent variable. The present study has sought to elucidate research inconsistencies associated with previous studies investigating potential links between ARHL and cognition in the following ways: firstly, there have been unresolved discrepancies regarding whether hearing loss clearly correlates to cognitive decline. This has been preliminarily reconciled after our statistical analysis. We also observed a trend of increased risk for cognitive impairment when extending follow-up. Secondly, CAD symptoms have not been well-considered in the hearing function-cognition relationship, as ARHL has appeared to be regarded in terms of mostly being peripheral hearing dysfunction^36^. However, from the evidence accumulated here, CAD has been shown to potentially increase the risk of cognitive impairment. Thirdly, we found that there was a dose-response association that more damaged peripheral hearing function contributed to a greater risk of cognitive impairment. Lastly, we statistically analyzed the influence of several potential confounding factors that previous studies did not cover. From the summarized data, we hope that our findings can help others understand the hearing function-cognition connection in older adults, in a clearer way. The possibility that hearing intervention may be a way to delay cognitive impairment could be further explored based on this relationship. This is especially essential because hearing rehabilitative interventions are largely underutilized^8,37^.

The relationship between hearing function and cognition in older adults has been observed since at least the 1960s. At that time, the first research about this connection found that subjects with organic mental syndrome (deficits in memory and intellect) had a higher prevalence of deafness^38^. The association has been seen in additional observational studies since then. A preliminary summarizing study^19^ reviewed existing literature to explore this relationship, despite the varied methods and limited sample sizes those investigations had applied. However, considering at least three opposing views^16–18^ exist within the preliminary study, it is difficult to conclude the presence of a solid correlation. Although the risk from hearing impairment on cognition cannot be addressed by randomized controlled trials (RCT) due to ethical concerns regarding possible harm to participants’ wellbeing (studies could not be designed to deliberately assign “hearing impairment” to the participants), recent well-designed cohort studies^14,20,24,29–31^ have emerged to serve as an alternative to explore the problem as thoroughly as possible. Still, some studies drew negative conclusions about the hearing function-cognition relationship^21,32,34^. Furthermore, few studies included the tests of central auditory function as part of the hearing evaluations, although CAD is prevalent (central auditory function declined with age in the elderly^39^, with the prevalence of 23–76.4%^40,41^); additionally, CAP and cognitive function are able to interact centrally (degraded speech communication in older people is partially influenced by additional central networks, including cognitive processing efforts^42^).

There are some hypothesized mechanisms that support the hearing function-cognition relationship. The frailty hypothesis states that ARHL is one of the markers of frailty – characterized by vulnerability to stressors – which could exert adverse health outcomes like cognitive frailty through inflammatory, vascular, hormonal, nutritional, and metabolic pathways^2,7,43^. The peripheral-central impairment hypothesis suggests that poor encoding of sound from the impaired cochleae has severe consequences including: demanding more cognitive resources for auditory perceptual processing, influencing brain structure, and reducing social engagement^44–49^. Moreover, some manifestations of CAD itself could be a sign of cognitive dysfunction^30^ (this has yet to be fully explored because some findings indicate that results of CAP tests are largely influenced by peripheral hearing function^50^, while others support the objectivity of the tests^51^). Additionally, the common factors hypothesis shows that hearing and cognitive disorders are correlated because they share similar neurodegenerative processes resulting from aging, vascular diseases, and oxidative stress^8,28,52^.

Our findings do have some limitations. Firstly, there were a limited number of the included studies, especially studies discussing CAD, which may make it difficult to show a conclusive connection. There were not enough studies for a publication bias analysis. Of the qualifying CAD-related studies, only two from the same research team were analyzed and only one CAP test was selected without further differential diagnoses to ensure CAD. Secondly, there was little data from cohorts with comprehensive controls including: ethnicity, gender, history of noise exposure, hereditary information, ototoxic drug use, lifestyle, socioeconomic status, and so on. This may impact the present findings because ARHL has been influenced by those factors^27,28^ although our analysis of some confounding variables did not result in significant heterogeneity.

Thirdly, the measures of cognition varied. Qualifying or quantifying instruments applied by the included studies incorporated the following classifications, diagnostic manuals, or screening tools: MMSE, Modified Mini-Mental State Examination (3MS), International Classification of Diseases (ICD), Cognitive Abilities Screening Instrument (CASI), DSM, and NINCDS-ADRDA. This could lead to potential heterogeneity for example, as screening tools such as MMSE, 3MS, and CASI neither play a role in diagnosing cognitive impairment (they do not have the same dementia definitions as the DSM^53,54^), nor do they predict mild cognitive impairment ultimately developing into dementia. Also, they cannot exclude cognitive difficulties from causes other than age-related cognitive decline^55–58^. Additionally, those cognition screening tools show a possible insufficient validity to find cognitive impairment across populations, compared with neuropsychological test batteries. For example, for ethnicity, MMSE had lower specificity within non-White groups^59^ despite previous evidence ascribing the ethnic differences to other causes^60,61^. Similarly, different norms of 3MS between White and Black populations may be worthy of consideration^62,63^. Therefore, though some studies^14,20,29^ ensured race was fully adjusted for in the cohorts, to eliminate possible bias, care should be applied when interpreting the cognitive function of the present included participants. Other limitations associated with evaluating cognitive status have included: a lack of studies which have extensively analyzed AD and other types of dementia as the outcomes, plus possible overestimation of cognitive impairment via verbal administration of some instruments and its dependence on preserved hearing function. The latter concern has been raised by previous studies^25,64,65^, although others^20,66^ have claimed that verbal administration is not an issue if questions are delivered by experienced examiners in quiet environments. Some studies have attempted to address this by cautiously applying multiple sources of data, and by adjusting total scores to account for any potential confounding aspects associated with sensory problems^33^. These aspects may make it difficult to reach a comprehensive conclusion until further studies emerge to clarify the field.

Fourthly, some combined studies^14,20,29^ presented potential factors likely contributing to relative heterogeneity; therefore, this may introduce bias. Lastly, the expected protective effect of hearing aid use was not significant in the results. This is perhaps because the included studies selected individuals from more hearing damaged populations, and there was an absence of variables like hearing aid fitting details, sufficiency in the hearing aid use, and other unmeasured factors^20^. Therefore, the potential effect of hearing aid use on cognition remains unknown. A well-designed RCT may help to determine the significance of hearing rehabilitation for cognitive decline, and findings from on-going studies^67–69^ will be eagerly awaited.

In conclusion, our findings suggest that older people with peripheral and central hearing impairment have a higher risk of cognitive impairment from a statistical perspective. With the limitations mentioned above, hopefully this preliminary finding will be strengthened by future studies.

## Methods

### Literature Search

According to the guidelines for the Preferred Reporting Items for Systematic Reviews and Meta-analyses (PRISMA)^70^ and the Meta-analysis of Observational Studies in Epidemiology (MOOSE)^71^, we searched the databases of PubMed and Embase from the inception of the databases indexed to December 2, 2016. Free text terms with the meaning of hearing loss without restriction were used: “auditory defect” or “auditory impairment” or “auditory dysfunction” or “deafness” or “deaf” or “hearing damage” or “hearing defect” or “hearing difficulty” or “hearing loss” or “hypacusia” or “hypacusis” or “impaired hearing” or “hearing impairment” or “hearing decline” or “hearing disability” or “poor hearing” or “presbycusis” or “hearing deficit” or “hearing trouble” or “hearing limitation” or “hearing handicap”. Mesh and Emtree words were also searched. The same strategies were applied to identify cognition disorder and dementia (the synonyms used for cognitive impairment and dementia were “cognition disorder” or “cognitive defect” or “cognitive deficit” or “cognitive disability” or “cognitive disorder” or “cognitive dysfunction” or “cognitive impairment” or “dementia” or “cognitive decline” or “cognitive difficulty” or “compromising cognition” or “cognitive compromise” or “cognitive trouble” or “troubled cognition” or “cognitive limitation” or “limited cognition” or “cognition limit” or “amentia” or “Alzheimer disease”). The age filters of the results were limited to middle-aged and aged individuals. For some major reviews^2,7,19^, we went through the contents and bibliographies, finding the related research by citation searching.

### Study selection

Two investigators (J.Y. and Y.S.) separately screened the studies. Duplicates were excluded; studies were then removed for reasons such as: having a sample size of less than 100 (in order to include high-quality studies in reference to a case-control study^72–74^), missing data, mean age at baseline not reaching 60 years, being irrelevant to our aim, and being non-observational studies. Next, the full texts of the remaining studies were reviewed to find: (1) prospective cohort studies; (2) extracted estimates were OR, RR, HR, or with enough data to calculate them, and their 95% CIs (odds ratio: ratio of the odds of cognitive impairment for the hearing impairment group to the odds of cognitive impairment for the control group; relative risk: ratio of hearing loss to cognitive impairment probability and normal hearing function to cognitive impairment probability; hazard ratio: the effect on the cognitive impairment rate of the difference between the hearing loss group and the control group estimated by the Cox hazards model^74,75^); (3) extracted results were possible to combine and adjusted with covariates. Divergences between the two investigators were discussed and resolved with the whole author group.

### Data extraction

We extracted the following characteristics from each study: (1) participants of the study; (2) baseline information of the study; (3) the measures employed to evaluate hearing and cognitive function; (4) adjusted variables; (5) follow-up; (6) adjusted estimates of OR, RR, and HR with 95% CIs. If necessary, authors of some included studies were contacted for more detailed information.

### Quality assessment

Quality assessment was made with standards from the Newcastle-Ottawa Scale (NOS)^76^. Selection (maximum 4 asterisks), comparability (maximum 2 asterisks), and outcome quality (maximum 3 asterisks) from the included studies were calculated. The scales were tabulated in values as the number of asterisks each study had gained. A maximum of 9 asterisks can be given for an individual study. The score for a high-quality study was defined as more than 5 asterisks^77^.

Two investigators (J.Y. and S.S.) carried out data extraction and quality assessment independently.

### Statistical analysis

Studies which had used OR or HR to calculate the incidence of cognitive disorder were pooled as approximate RR because hearing loss resulting in cognitive impairment is a rare event. Meta-analysis was conducted with the inverse variance (IV) method. In the studies including covariates, we extracted the most comprehensively adjusted estimates. Heterogeneity was explored with the Cochrane *Q*-statistic among studies: *P*_heterogeneity_ < 0.10 and *I*^2^ > 50% indicating significant heterogeneity^78–80^. In our analyses, we used the random-effect models because there was variation among these studies in characteristics like sample size, follow-up, population characteristics, and definitions for cognitive impairment. We estimated the dose-response trend based on risks calculated by categories of peripheral hearing function. Covariates as potential confounding factors were retrieved and analyzed in groups with meta-regression. A confounding factor was recognized with a meta-regression *P* < 0.05^81^. Sensitivity analysis was conducted by the one-study-out method: omitting one study at a time, sequentially, to test the combined RR from the remaining studies so as to see the stableness of each meta-analysis^82^.

Meta-analysis was performed by Cochrane Review Manager (RevMan, Version 5.3. Copenhagen: The Nordic Cochrane Centre, The Cochrane Collaboration, 2014) and Stata (MP 14.1, Stata Corp, College Station, TX, USA). Statistical significances were set at *P* < 0.05 for all the analyses unless otherwise specified.

## Electronic supplementary material

Supplementary Information
