# Supplementary material for: The risk of cognitive impairment associated with hearing function in older adults: a pooled analysis of data from eleven studies
Source: Sci Rep. 2018 Feb 1;8:2137. doi: 10.1038/s41598-018-20496-w (PMC5794920; doi:10.1038/s41598-018-20496-w)
Supplement: Supplementary file 1 — Supplementary Information [file 41598_2018_20496_MOESM1_ESM.pdf]

Supplementary Information

The risk of cognitive impairment associated with hearing function in older adults: a pooled analysis of data from eleven studies

Jing Yuan[1], Yu Sun[1], Shuping Sang[2], Jessica Huynh Pham[3], Wei-Jia Kong[1,*]

[1]Department of Otorhinolaryngology, Union Hospital, Tongji Medical College, Huazhong University of Science and Technology, Wuhan, Hubei 430022, PR China.

[2]School of Medicine, Yunnan University, Kunming, Yunan 650031, PR China

[3]School of Medicine, Case Western Reserve University, Cleveland, Ohio 44106, United States

*entwjkong@hust.edu.cn

Studies	Representativeness of the hearing-impaired cohort	Selection of the controlled cohort	Hearing evaluations	No dementia at the baseline	Comparability	Cognitive status	Follow-up length	Adequacy of follow-up	Total
Deal JA, 2017[1]	*	*	*	*	**	*	*	No*	8*
Fritze T, 2016[2]	*	*	*	*	**	*	*	NA	8*
Gates GA, 2011[3]	*	*	*	*	*	*	No*	NA	6*
Gates GA, 1996[4]	*	*	*	*	*	*	*	No*	7*
Gurgel RK, 2014[5]	*	*	No*	*	**	*	*	NA	7*
Hong T, 2016[6]	*	*	*	NA	*	*	*	No*	6*
Karpa MJ, 2010[7]	*	*	*	No*	*	*	*	NA	6*
Lin FR, 2013[8]	*	*	*	*	**	*	*	NA	8*
Lin FR, 2011[9]	No*	*	*	*	**	*	*	NA	7*
Lin MY, 2004[10]	No*	*	*	NA	**	*	No*	*	6*
Lyu J, 2016[11]	*	*	No*	*	No*	*	*	*	6*

Supplementary Table S1. Quality assessment of included studies (Newcastle-Ottawa Scale). Study quality was valued as the number of stared requirements each study met: **=met two stared requirements in this category, *=met one stared requirement in this category, No*=did not meet any stared requirement, NA=information not available.

Studies	Estimates	ES lower 95%CI	ES upper 95% CI	Ear sides (PTA)	Follow-up	Ethnicity	Gender	Adjustment	Hearing evaluations	Instruments for cognitive status
Deal JA, 2017[1]	1.55	1.10	2.19	1	2	1	1	1	1	1
Deal JA, 2017[1]	1.64	1.16	2.30	1	2	1	1	3	1	1
Deal JA, 2017[1]	1.78	1.32	2.39	1	2	1	1	2	1	1
Fritze T, 2016[2]	1.01	0.89	1.15	4	1	1	1	1	2	2
Fritze T, 2016[2]	1.03	0.84	1.26	4	1	1	1	3	2	2
Gates GA, 1996[4]	0.90	0.49	1.66	1	1	1	1	3	1	1
Gates GA, 1996[4]	1.03	0.74	1.45	2	1	1	1	3	1	1
Gurgel RK, 2014[5]	1.28	1.04	1.57	5	2	1	1	3	2	2
Gurgel RK, 2014[5]	1.27	1.03	1.56	5	2	1	1	1	2	2
Hong T, 2016[6]	2.08	0.46	9.43	1	2	1	1	3	1	1
Hong T, 2016[6]	1.04	0.22	4.93	1	1	1	1	3	1	1
Hong T, 2016[6]	1.02	0.61	1.70	2	1	1	1	3	1	1
Hong T, 2016[6]	1.09	0.65	1.82	2	2	1	1	3	1	1
Lin FR, 2013[8]	1.36	1.08	1.70	1	1	1	1	1	1	1
Lin MY, 2004[10]	1.38	0.95	2.00	1	1	2	3	1	1	1
Lin MY, 2004[10]	1.64	1.37	1.97	1	1	2	3	2	1	1
Lyu J, 2016[11]	1.36	0.98	1.90	5	1	3	1	2	2	1
Lyu J, 2016[11]	1.94	1.29	2.92	5	1	3	3	2	2	1
Lyu J, 2016[11]	0.87	0.54	1.39	5	1	3	2	2	2	1

Supplementary Table S2. Original estimates for meta-regression. Dummy variables: ear sides from PTA (better ear=1, worse ear=2, two ears=3, one ear=4, unspecified=5), maximum follow-up (≤6 years=1, >6 years=2), ethnicity (mixed cohort=1, Black-excluded cohort=2, Asian cohort=3), gender (mixed cohort=1, Male cohort=2, Female cohort=3), level of adjustment (fully adjustment=1, no adjustment=2, partial adjustment=3), hearing evaluations (pure-tone audiometry=1, others=2), instruments for cognitive status (MMSE or revised=1, others=2). Abbreviations: CI=confidence interval, ES=estimate, PTA=pure-tone average, MMSE=Mini-Mental State Examination.

References

1. Deal, J. A. *et al.* Hearing impairment and incident dementia and cognitive decline in older adults: The Health ABC Study. *J Gerontol A Biol Sci Med Sci.* **72,** 703-709 (2017).
2. Fritze, T. *et al.* Hearing impairment affects dementia incidence. An analysis based on longitudinal health claims data in Germany. *PLoS One.* **11,** e0156876 (2016).
3. Gates, G. A., Anderson, M. L., McCurry, S. M., Feeney, M. P. & Larson, E. B. Central auditory dysfunction as a harbinger of Alzheimer dementia. *Arch Otolaryngol Head Neck Surg.* **137,** 390-395 (2011).
4. Gates, G. A. *et al.* Central auditory dysfunction, cognitive dysfunction, and dementia in older people. *Arch Otolaryngol Head Neck Surg.* **122,** 161-167 (1996).
5. Gurgel, R. K. *et al.* Relationship of hearing loss and dementia: A prospective, population-based study. *Otol Neurotol.* **35,** 775-781 (2014).
6. Hong, T., Mitchell, P., Burlutsky, G., Liew, G. & Wang, J. J. Visual impairment, hearing loss and cognitive function in an older population: Longitudinal findings from the Blue Mountains Eye Study. *PLoS One.* **11,** e0147646 (2016).
7. Karpa, M. J. *et al.* Associations between hearing impairment and mortality risk in older persons: The Blue Mountains Hearing Study. *Ann Epidemiol.* **20,** 452-459 (2010).
8. Lin, F. R. *et al.* Hearing loss and cognitive decline in older adults. *JAMA Intern Med.* **173,** 293-299 (2013).
9. Lin, F. R. *et al.* Hearing loss and incident dementia. *Arch Neurol.* **68,** 214-220 (2011).
10. Lin, M. Y. *et al.* Vision impairment and combined vision and hearing impairment predict cognitive and functional decline in older women. *J Am Geriatr Soc.* **52,** 1996-2002 (2004).
11. Lyu, J. & Kim, H.-Y. Gender-specific incidence and predictors of cognitive impairment among Older Koreans: Findings from a 6-year prospective cohort study. *Psychiatry Investig.* **13,** 473 (2016).
